# Benchmarking Fast Healthcare Interoperability Resources–Based Analytics: Quantitative Study of RESTful Server Queries and Big Data Engines

**DOI:** 10.2196/82924

**Published:** 2026-07-17

**Authors:** Christian Gulden, Marvin Kampf, Detlef Kraska, John Grimes, Thomas Ganslandt, Hans-Ulrich Prokosch, Susanne A. Seuchter, Jonathan M. Mang, Peter Pallaoro, Paul-Christian Volkmer, Jasmin Ziegler

**Affiliations:** 1 Lehrstuhl für Medizinische Informatik Institut für Medizininformatik, Biometrie und Epidemiologie Friedrich-Alexander-Universität-Erlangen-Nürnberg Erlangen Germany; 2 Bavarian Cancer Research Center (BZKF) Erlangen Germany; 3 Medical Center for Information and Communication Technology Universitätsklinikum Erlangen Erlangen Germany; 4 CSIRO Health and Biosecurity Australian e-Health Research Centre Brisbane Australia; 5 Chair of Medical Informatics, Institute for AI and Informatics in Medicine (AIIM), TUM University Hospital TUM School of Medicine and Health Technical University of Munich Munich Germany; 6 Data Integration Center, TUM University Hospital TUM School of Medicine and Health Technical University of Munich Munich Germany; 7 Anneliese Pohl Krebszentrum Marburg Comprehensive Cancer Center Universitätsklinikum Gießen und Marburg Marburg Germany

**Keywords:** Fast Healthcare Interoperability Resources, FHIR, benchmark, performance, big data

## Abstract

**Background:**

Electronic health records offer vast clinical data for health care research, but interoperability challenges often hinder comprehensive analysis. The Health Level Seven Fast Healthcare Interoperability Resources (FHIR) standard addresses these challenges, although its nested and interconnected resource format can be complex for analytics. Several tools have emerged to facilitate analytical access, either by querying FHIR servers via representational state transfer (REST) APIs or encoding resources in relational formats. However, the performance implications of these methods remain largely unexplored.

**Objective:**

This study aimed to benchmark the performance characteristics of different FHIR-based analytical approaches comparing REST API queries against SQL- and Spark-based big data frameworks operating on FHIR-encoded data.

**Methods:**

We benchmarked the FHIR-PYrate library, which interfaces with a FHIR server’s REST API, against Pathling, a library built for analytics based on Apache Spark, and Trino, a general-purpose SQL query engine. We defined and implemented multiple queries in each engine using 3 common analytics scenarios—data aggregation, counting, and extraction. Execution times were measured across Synthea-generated datasets of increasing size.

**Results:**

On the largest dataset, containing 71,285,064 FHIR resources, Trino completed the aggregate query more than 12,000 times faster, and Pathling did so approximately 500 times faster than FHIR-PYrate. On average across all queries, Trino outperformed FHIR-PYrate, executing extraction queries 33 times faster and count queries 1.8 times faster. Pathling achieved a 2.6-time speedup for extraction queries, but FHIR-PYrate was approximately 13 times faster for count queries.

**Conclusions:**

While the REST-based FHIR search API is useful for standard queries and retrieving specific patient records and can outperform alternatives for some count queries, it generally lacks the performance and expressiveness needed for complex analytics. In contrast, alternative engines such as Trino and Pathling demonstrated substantial performance advantages for these scenarios.

## Introduction

Electronic health records provide digital means to store and retrieve clinical data for millions of patients. Leveraging these real-world data provides an exciting opportunity for health care research [1]. However, the heterogeneity of electronic health record systems often results in interoperability challenges, hindering comprehensive analysis—especially across institutional boundaries [2,3]. This is where the Health Level Seven Fast Healthcare Interoperability Resources (FHIR) can help by providing a standardized framework for modeling and exchanging health care data [4]. The standard describes a data model consisting of resources that represent concepts in the health care domain. These resources are connected via references and form a graph. For example, a FHIR “Condition” resource representing a medical diagnosis may reference a “Patient” resource to which it applies and a “Practitioner” resource identifying the person recording the diagnosis. The FHIR standard also defines an HTTP representational state transfer (REST) interface and its semantics. This interface is implemented by FHIR servers to support the creation, reading, updating, and deletion of resources. Additionally, FHIR Search provides a comprehensive framework for filtering and retrieving relevant resources [5].

The increased adoption of FHIR is driving its use for analytic use cases, including decision support, feasibility research, and epidemiological research [6,7]. When conducting analytical research on FHIR data, familiarity with the graphlike structure, conventions, data types, and search semantics is required. From a purely syntactic perspective, data science and machine learning are usually conducted on tabular data, whereas FHIR resources are nested and interconnected—often encoded as JSON or XML file formats. Due to this gap, several projects aim to convert FHIR resources to a structure more suitable for analytical use cases. These approaches either interact with FHIR servers in a combination of FHIR Search and local postprocessing [8-11] or encode and store resources in a relational or columnar format first [12-15]. Nevertheless, the performance implications of these methods remain largely unexplored.

In this work, we compared analytical approaches that operate on Health Level Seven FHIR as the underlying data representation but differ in how the data are accessed and processed, ranging from REST-based querying of FHIR servers to SQL- and Spark-based querying of tabular-encoded FHIR datasets.

## Methods

### Engines and Queries

We compared the performance of 3 different approaches for conducting analytics on persisted FHIR resources:

- FHIR-PYrate (version 0.2.3) [9]: a Python library that interacts with a FHIR server’s REST API to filter and download resources and with FHIRPath [16] to extract individual attributes
- Pathling (version 7.2.0) [12]: a Python, Scala, Java, and R library used to query FHIR data previously encoded as Apache Spark [17,18] datasets; like FHIR-PYrate, it allows for the use of FHIRPath to describe the tabular data to be extracted from the resources
- Trino (version 478) [19]: a distributed American National Standards Institute (ANSI) SQL-compliant federated query engine

The above-mentioned engines were chosen to allow for comparison between accessing resources directly via a FHIR server’s search features, via a more FHIR-native way using FHIRPath expressions, and via SQL—often considered the lingua franca of data analytics [20]. Comparing Pathling and Trino additionally allowed us to benchmark 2 frameworks based on the same Delta Lake table format [21] but using different query engines. This storage-compute decoupling approach is increasingly adopted in modern data warehouse designs [22,23].

We compared the performance of each framework in 3 common analytics scenarios:

- Aggregation: summarizing data to provide statistics such as mean, median, minimum, maximum, sum, and count of values
- Counting: counting the number of resources that satisfy given criteria (eg, this is a common use in feasibility studies to assess the number of potentially eligible patients)
- Extraction: retrieving a subset of relevant data in a tabular format filtered to answer a research question

For the aggregation scenario, we counted the number of FHIR “Observation” resources grouped by their code (eg, the total number of hemoglobin level observations across all patients). This is a common query for inventorying and presenting total available data, such as in a dashboard. As FHIR Search lacks built-in aggregation capabilities, the implementation using FHIR-PYrate first had to download all “Observation” resources into an in-memory pandas DataFrame and execute the aggregation using the pandas “groupby” function. Therefore, this query represents a fundamentally challenging scenario for standard-based FHIR Search interfaces as the FHIR Search specification does not define native server-side aggregation operations. The performance characteristics are determined by the need for client-side retrieval and processing of all available resources.

For both counting and extracting, we implemented 3 queries to retrieve data. The queries were chosen to be close to real-world use cases while at the same time being compatible with the synthetic data against which they were executed. Further, the queries were increasingly complex, integrating multiple resource types and adding multiple filter conditions. The queries were also created in a way that would allow them to be represented as a single FHIR Search request. This made comparison between the engines easier as no additional postprocessing had to take place inside the benchmarking code.

The descriptions of the 3 queries are provided in Table 1. For the count scenario, only the number of patients satisfying the filter criteria was returned, whereas for the extraction and aggregation scenarios, a projection of resource elements in tabular form was written to a CSV file.

**Table 1:** Description of the 3 queries used to benchmark the engines for the count and extraction scenarios.

Query name	Description
Gender-age	All patients whose gender was set to “female” and whose birth date was on or after January 1, 1970
Diabetes	All patients born after January 1, 1970, with a diagnosis of diabetes documented for an encounter taking place on or after January 1, 2020
Hemoglobin	All patients with a hemoglobin laboratory value given either in mass per volume format that was higher than 25 g/dL or as a relative percentage of HbA_1c_^a^ compared to total blood hemoglobin that was higher than 5%

^a^HbA_1c_: hemoglobin A_1c_.

The main difference between the count queries and the aggregation and extraction ones is that the former only returns a single scalar result value to the caller, so the impact of large data transfers between the benchmark client and the query implementation is significantly reduced.

To compare the scalability of the different engines across all scenarios, we executed the aggregate, count, and extract queries on progressively larger FHIR datasets generated using Synthea [24]. The Synthea command-line tool was used to generate a reproducible dataset of 1000, 5000, 10,000, 50,000, and 100,000 records. These resources were stored as JSON-encoded files in the local file system. For each record count, the scripted benchmarking workflow was as follows:

- Start all the required software components.
- Encode the FHIR resources as Delta Lake tables using the Pathling server’s bulk import operation.
- Run Delta Lake–specific OPTIMIZE and VACUUM commands to optimize the file layout.
- Load the synthetic data into the Blaze FHIR server followed by the HAPI FHIR server.
- Run PostgreSQL’s VACUUM FULL and ANALYZE commands against the largest HAPI FHIR PostgreSQL tables.
- Stop the Pathling server as it is only used for encoding and persisting the resources in MinIO object storage as tabular data.
- For each query type (count, extract, and aggregate), repeat the following process 10 times: (1) record the total time taken per engine and query from the moment the query is submitted until the CSV file is fully written to disk; (2) execute the SQL queries against Trino consecutively, download all results, and save them as a CSV file; (3) restart the Trino container and MinIO and wait 30 seconds for start-up to complete to ensure that the system stabilizes and background tasks are complete before measurements begin; (4) execute the queries using Pathling and save them as a CSV file; (5) reset the cache, force garbage collection, reinitialize Spark, and wait 30 seconds; (6) execute the queries using FHIR-PYrate, saving the results as CSV files; and (7) restart the FHIR server container and wait 30 seconds.

Restarting the FHIR server and Trino clears in-memory caches and frees up unused memory for the operating system. Each benchmark run started with a different query in a round-robin fashion, ensuring that no query consistently preceded others and minimizing potential order effects. The workflow was implemented using Python (version 3.12; Python Software Foundation), and runtimes were measured using a monotonic performance counter. The benchmarks were executed on a single virtual machine (type CCX53) hosted on Hetzner Cloud running Ubuntu (version 24.04; Canonical Ltd). The machine was equipped with 32 dedicated AMD EPYC (Milan) virtual central processing units (CPUs), 128 GB of RAM, and a 600-GB Non-Volatile Memory Express solid-state drive.

**Figure 1:**
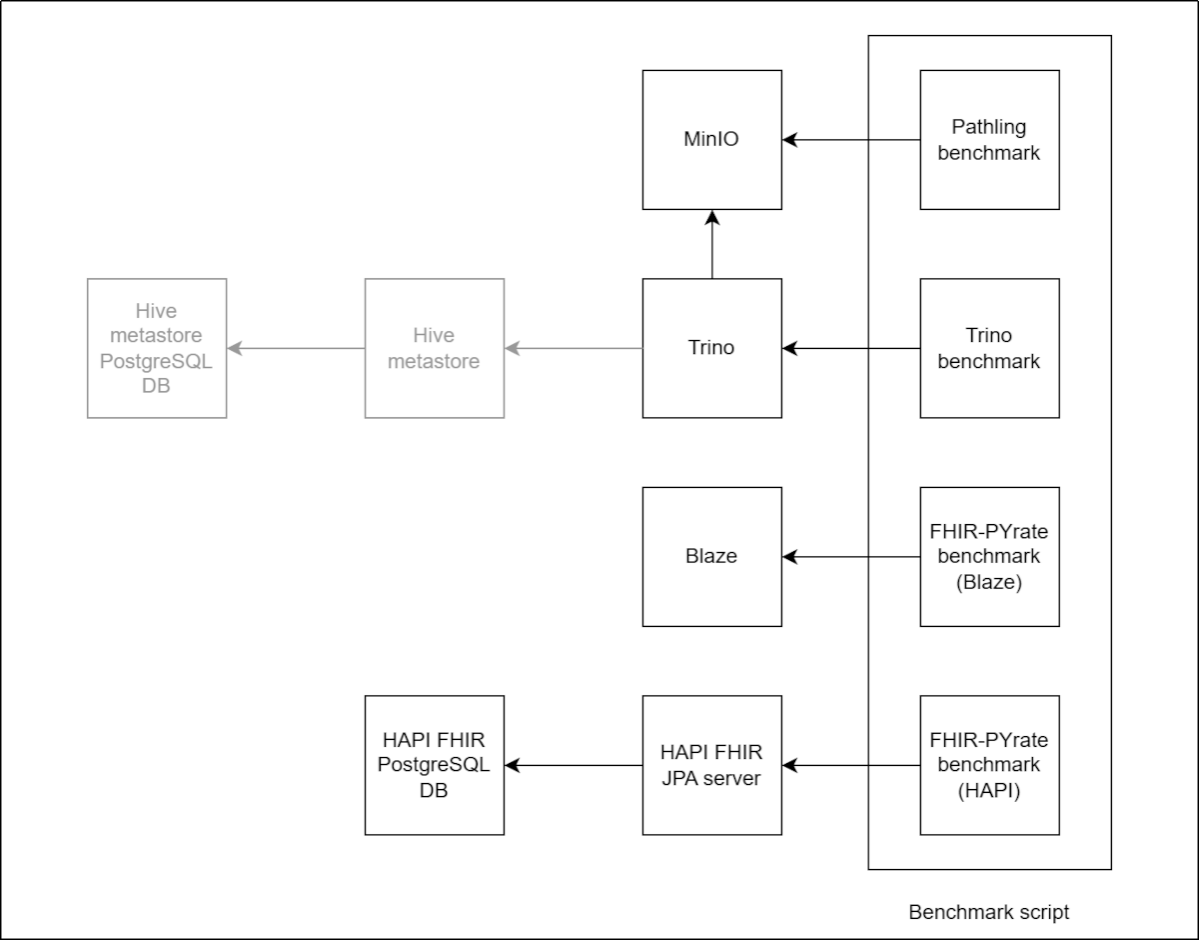
Application configuration of the benchmarking virtual machine. Both the Hive metastore and the Hive metastore PostgreSQL DB were not on the critical benchmark data path and are drawn in lighter gray. FHIR: Fast Healthcare Interoperability Resources.

Figure 1 shows the application setup on the virtual machine. For the Trino benchmark, a Hive metastore (The Apache Software Foundation) was used to register the resource tables and their location in the MinIO object storage. However, the metastore was not part of the critical path for the benchmark and was only used by Trino to determine the physical location of the Delta Lake tables.

The individual application container images and versions are listed in Table 2. The table also includes the memory limit set for each application: for Trino, Blaze, and the HAPI FHIR JPA server, the memory limits were set via the “-Xmx64g” Java Virtual Machine option. For the remaining applications, the memory limits were set using group memory limits on the containers [25].

**Table 2:** Applications and their respective container image, version, and memory limit used for the benchmark.

Application	Container image	Version	Memory limit
Trino	docker.io/trinodb/trino	478	64 GB
Blaze	docker.io/samply/blaze	1.2.0	64 GB
Pathling server	docker.io/aehrc/pathling	7.2.0	56 GB
MinIO	docker.io/minio/minio	RELEASE.2025-09-07T16-13-09Z	8 GB
Hive metastore	docker.io/apache/hive	4.0.0	2 GB
Hive metastore Database	docker.io/library/postgres	18.1	1 GB
HAPI FHIR^a^ server	docker.io/hapiproject/hapi	8.6.0-1	48 GB
HAPI FHIR server Database	docker.io/library/postgres	18.1	16 GB

^a^FHIR: Fast Healthcare Interoperability Resources.

The Blaze server was tuned following the production configuration guide [26], and the HAPI FHIR server PostgreSQL database was configured using the PGTune online tool [27] for data warehouse workloads. For Pathling, we deviated from the default configuration by setting the Parquet compression codec to Zstandard level 9 and the serializer to KryoSerializer. The complete benchmark configuration is available in the public source code repository.

### Measuring Resource Use for Data Loading

As a first step, the FHIR resources generated by the Synthea command-line interface had to be loaded into the FHIR servers (for HAPI and Blaze) or encoded as Delta Lake tables (for Pathling and Trino). To load the resources into the servers, we used blazectl (version 1.2.0) with client concurrency set to 32; for Delta Lake, we used the Pathling server’s FHIR bulk import API. The former tool reports the import duration after completion; for the latter, we used the Unix time binary to measure the duration of the synchronous import triggered via curl.

We used cAdvisor (version 0.53.0) to record the container’s CPU and memory use during the data loading. The metrics were stored in a Prometheus time-series database (version 3.7.3), retrieved when the imports finished, and stored in a CSV file for later visualization. Prometheus was configured to scrape cAdvisor metrics every 5 seconds.

Per-container disk use was measured after the import was completed using the Docker command-line interface.

### Comparing FHIR Server Performance

A separate benchmark was conducted to compare the performance of the 2 FHIR server implementations: Blaze and the HAPI FHIR server. The original hemoglobin query, which included multiple OR-combined composite code-value-quantity filters, could not be executed on the HAPI FHIR server due to time-out errors starting on the smallest record count. Therefore, a simplified hemoglobin query variant was used for the server-to-server benchmark that only contained one of the OR-combined filters. As the HAPI FHIR server’s PostgreSQL database volume size exceeded the available disk space for the record count of 100,000, we excluded it from those benchmarks. The runs were repeated 10 times for the record counts, but only 3 times for the final 50,000 records due to the excessive runtime.

### Measuring Cache Impact

The benchmarks so far were constructed to measure worst-case cold-start performance by repeatedly restarting the services between runs. To assess the impact of caching, we repeated the count and extracted workload benchmarks for the largest record count without restarting the services. The runs were repeated 5 times following 1 warm-up run.

### Measuring Data Skew Impact

To evaluate robustness to realistic data skew, we ran another benchmark using three new count queries based on the distribution of “Observation” codes in the Synthea dataset for the largest record count: (1) skewed-hot codes, which filter on 5 high-frequency “Observation” codes selected from the 10 most common codes; (2) skewed-rare codes, which filter on 5 low-frequency “Observation” codes selected from the 10 rarest codes; and (3) skewed-mixed codes, which combine both the hot and rare codes using the logical OR operator.

These workloads stress low-selectivity scans vs highly selective filters without modifying the dataset. Each query was executed 5 times, and we measured the mean of the execution time.

## Results

### Overview

Tables 3 and 4 detail the number of resources corresponding to the Synthea record counts, the size of the raw JSON files, the time taken to import the resources to the FHIR servers and Delta Lake, and the size of the volumes used to store the imported resources.

**Table 3:** Fast Healthcare Interoperability Resources (FHIR) resource counts, file size, import duration, and volume sizes corresponding to the Synthea record counts. The HAPI FHIR server was excluded from the largest record count benchmarks as the required disk space would exceed the available resources.

Records, n	FHIR resources by type, n						Synthea JSON file size	
	Patient	Condition	Observation	Encounter	Total	Bulk		Transaction
1000	1144	38,516	515,968	62,476	618,104	653 MB		1018 MB
5000	5760	202,166	2,963,729	339,867	3,511,522	3.6 GB		5.6 GB
10,000	11,481	402,577	5,915,941	683,151	7,013,150	7.2 GB		12 GB
50,000	57,538	2,047,676	30,371,746	3,475,991	35,952,951	37 GB		57 GB
100,000	115,153	4,075,593	60,168,356	6,925,962	71,285,064	73 GB		113 GB

**Table 4:** Import duration and volume sizes corresponding to the Synthea record counts. The HAPI Fast Healthcare Interoperability Resources (FHIR) server was excluded from the largest record count benchmarks as the required disk space would exceed available resources.

Records, n	Import duration				Volume size		
	Pathling	Blaze server	HAPI server	MinIO (Pathling and Trino)		Blaze	HAPI PostgreSQL database
1000	1 min 26 s	2 min 56 s	5 min 12 s	103.5 MB		3.924 GB	19.3 GB
5000	5 min 52 s	14 min 52 s	25 min 25 s	584.1 MB		7.729 GB	47.91 GB
10,000	10 min 50 s	26 min 16 s	52 min 23 s	1.177 GB		11.33 GB	78.53 GB
50,000	47 min 44 s	2 h 12 min 49 s	4 h 37 min 53 s	6.386 GB		44.19 GB	379.2 GB
100,000	1 h 26 min 27 s	4 h 22 min 49 s	N/A^a^ (excluded)	11.35 GB		84.05 GB	N/A (excluded)

^a^N/A: not applicable.

Because of the way Synthea was implemented, specifying a record count did not exactly return the same number of “Patient” resources. In the following graphs, we refer to the dataset size as “record count,” corresponding to the individual resource counts in Tables 3 and 4.

**Figure 2:**
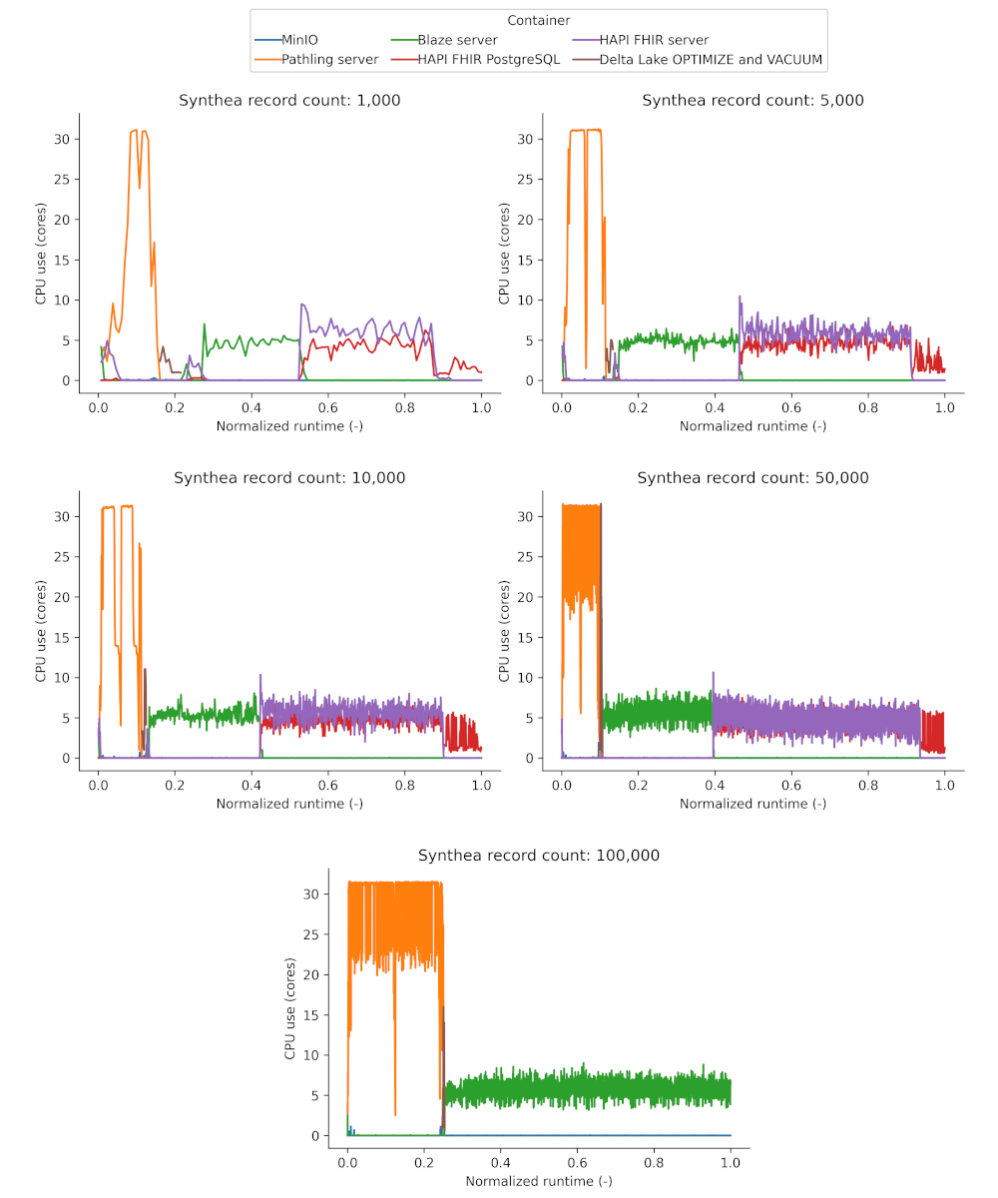
Central processing unit (CPU) use of the containers involved in loading the Fast Healthcare Interoperability Resources (FHIR) resources from the local file system into the servers and object storage.

**Figure 3:**
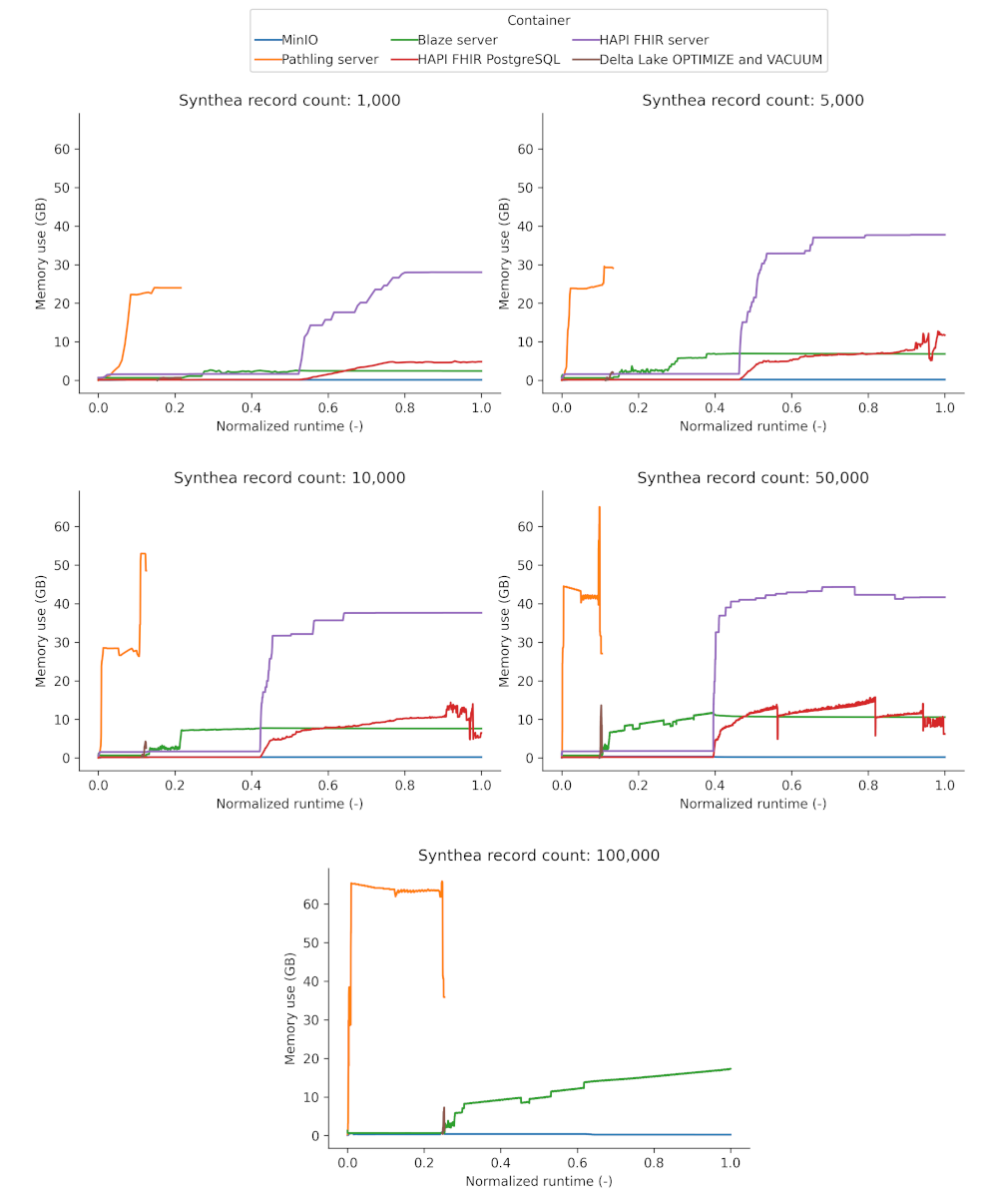
Memory use of the containers involved in loading the Fast Healthcare Interoperability Resources (FHIR) resources from the local file system into the servers and object storage.

Figures 2 and 3 show the memory and CPU use during data loading across the evaluated systems. During the bulk import into the Pathling server, memory consumption rapidly reached the configured limits, and CPU use became saturated, particularly for larger record counts. After completion of the import, the execution of the Delta Lake OPTIMIZE and VACUUM commands resulted in a brief peak in both CPU and memory use due to the deletion of expired files from object storage.

In contrast, the Blaze server exhibited lower CPU and memory use during transactional ingestion of FHIR resources. For the HAPI FHIR setup, CPU use was split between the PostgreSQL database and the server application, whereas memory consumption was dominated by the server. Following import, the execution of VACUUM and ANALYZE statements led to increased CPU and memory use on the PostgreSQL database, which was visible toward the end of the runtime.

### FHIR Server Performance

**Figure 4:**
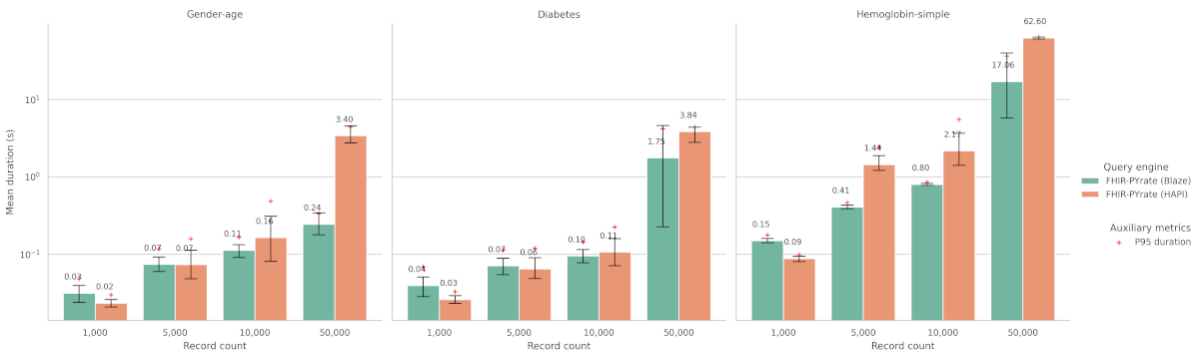
Mean execution times (log-scale; lower is better) by query and record count for the HAPI Fast Healthcare Interoperability Resources and Blaze servers for count queries. Error bars indicate the 95% CIs of the mean. The P95 duration is indicated by a red cross. For the largest record count, runs were repeated 3 times; for all other record counts, runs were repeated 10 times.

**Figure 5:**
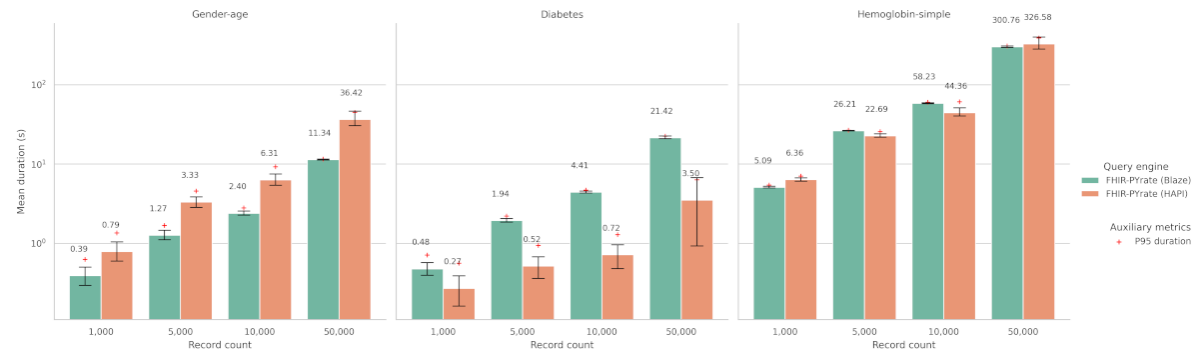
Mean execution times (log-scale; lower is better) by query and record count for the HAPI Fast Healthcare Interoperability Resources and Blaze servers for extraction queries. Error bars indicate the 95% CIs of the mean. The P95 duration is indicated by a red cross. For the largest record count, runs were repeated 3 times; for all other record counts, runs were repeated 10 times.

**Figure 6:**
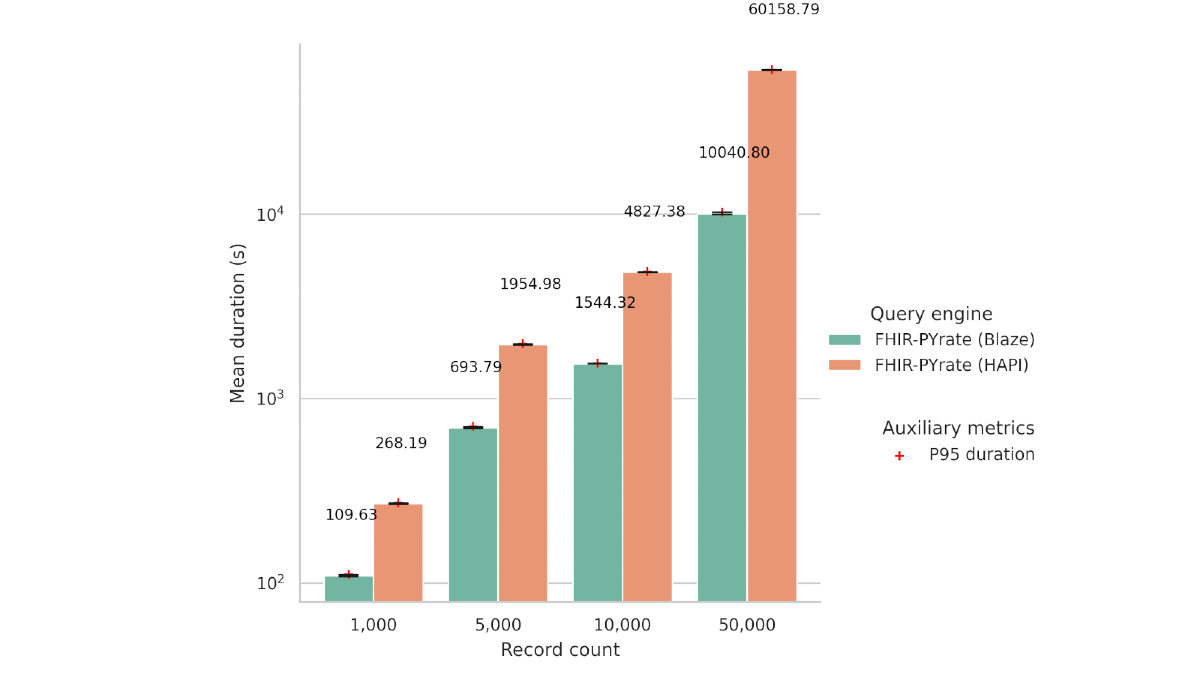
Mean execution times (log-scale; lower is better) by query and record count for the HAPI Fast Healthcare Interoperability Resources and Blaze servers for aggregation queries. Error bars indicate the 95% CIs of the mean. The P95 duration is indicated by a red cross. For the largest record count, runs were repeated 3 times; for all other record counts, runs were repeated 10 times.

Figure 4 shows the duration of the count queries comparing Blaze and the HAPI FHIR server. For smaller record counts and the 2 simpler gender-age and diabetes queries, performance was similar. At the largest record counts, and across all queries, Blaze completed the requests faster. For the data extraction scenario shown in Figure 5, results varied depending on the query: Blaze exhibited lower execution time for gender-age extraction but performed worse for the diabetes query. Both servers demonstrated similar performance for the hemoglobin query. Figure 6 shows the results of data aggregation queries. Across all record counts and queries, execution times were lower for Blaze than for HAPI FHIR.

### Cross-Framework Performance Comparison

**Figure 7:**
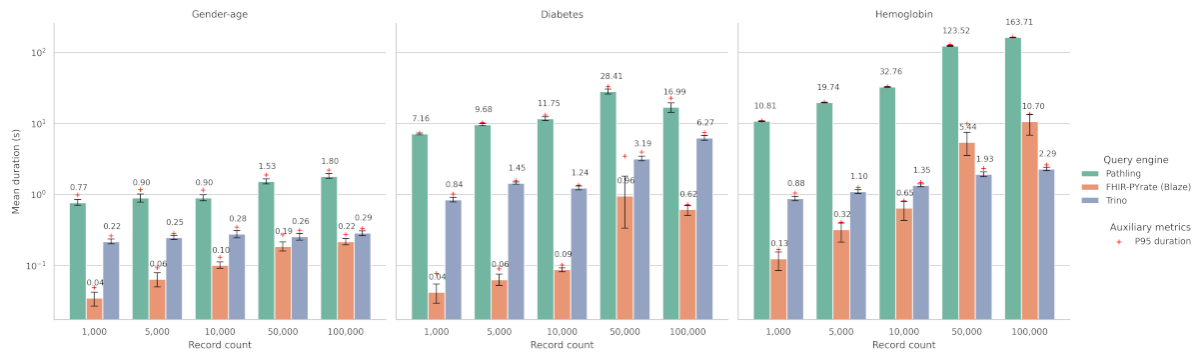
Mean execution times (log-scale; lower is better) by query and record count comparing Pathling, Blaze, and Trino for count queries. Error bars indicate the 95% CIs of the mean. All runs were repeated 10 times.

For count workloads, Blaze exhibited subsecond execution times for the gender-age and diabetes queries across all record counts (Figure 7), outperforming both Trino and Pathling. For the complex hemoglobin query, Trino performed best for the largest record counts.

**Figure 8:**
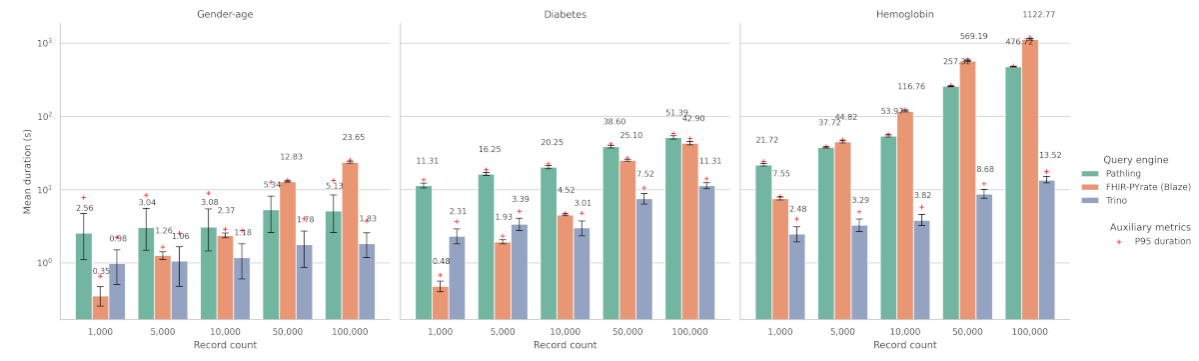
Mean execution times (log-scale; lower is better) by query and record count comparing Pathling, Blaze, and Trino for extraction queries. Error bars indicate the 95% CIs of the mean. All runs were repeated 10 times.

For extraction workloads (Figure 8), Trino maintained the lowest mean latency for the largest record counts across all queries. FHIR-PYrate exhibited linear growth and substantially higher absolute latency at larger record counts, exceeding 1000 seconds at 100,000 records. At smaller sizes, it outperformed both Pathling and Trino. Pathling showed similar linear growth to that of FHIR-PYrate.

**Figure 9:**
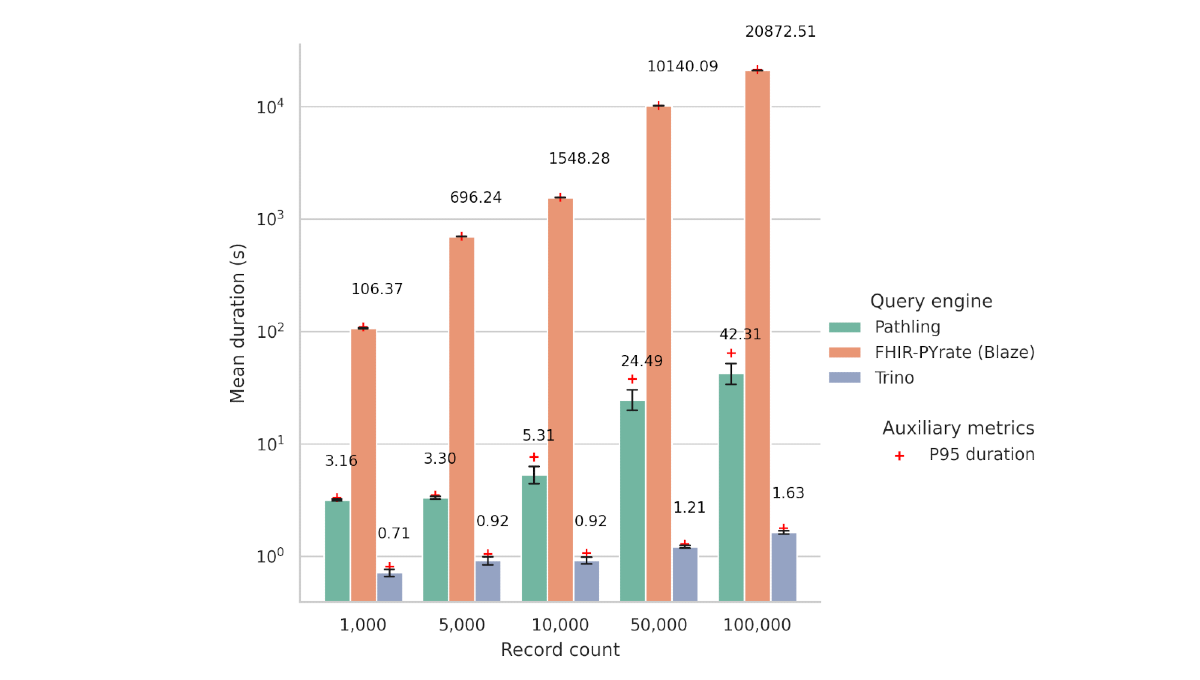
Mean execution times (log-scale; lower is better) by query and record count comparing Pathling, Blaze, and Trino for aggregation queries. Error bars indicate the 95% CIs of the mean. All runs were repeated 10 times.

Finally, the aggregate workload (Figure 9) amplified the performance differences observed in extraction queries. Trino again showed the lowest latency and the most stable scaling behavior across record counts. Pathling scaled linearly but with higher absolute runtimes than Trino. FHIR-PYrate showed the steepest performance degradation, with aggregate queries reaching multi-hour execution times at the largest record count.

### Cache Impact

**Figure 10:**
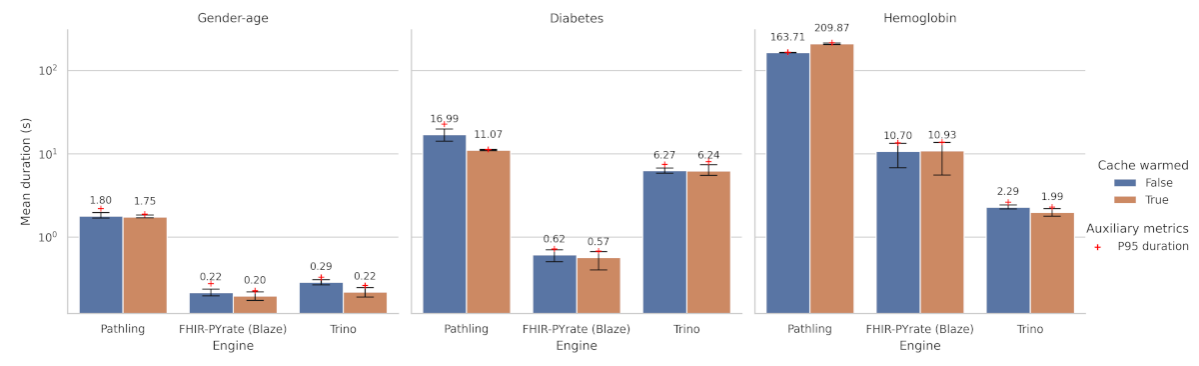
Log-scaled mean execution time for the count queries of 5 consecutive runs (lower is better) comparing warm-cache to cold-cache runtimes. Error bars indicate the 95% CIs of the mean.

**Figure 11:**
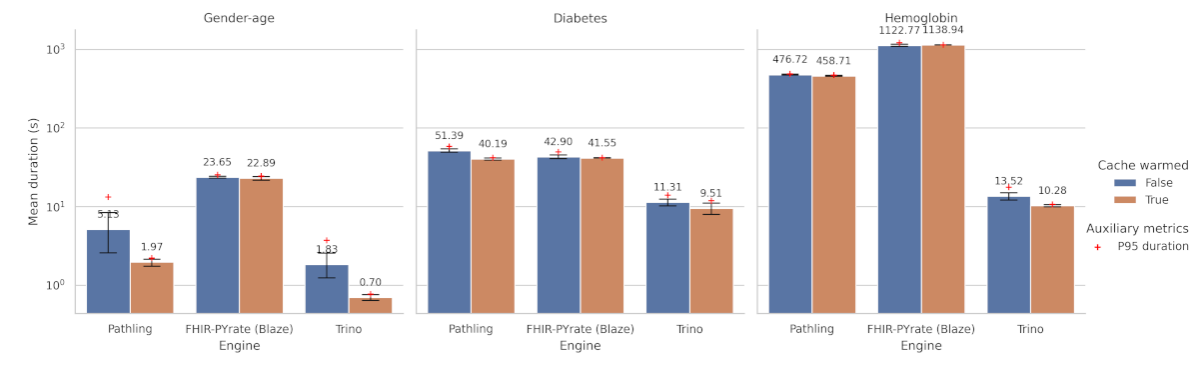
Log-scaled mean execution time for the extraction queries of 5 consecutive runs (lower is better) comparing warm-cache to cold-cache runtimes. Error bars indicate the 95% CIs of the mean.

Figures 10 and 11 show the impact of keeping services running for both the count (Figure 10) and extraction (Figure 11) query types. Cache warming had a small effect on count-based queries across all engines, whereas extraction workloads showed lower runtimes under warm-cache conditions for Pathling and Trino. The FHIR back end showed smaller differences between cold and warm execution.

### Data Skew

**Figure 12:**
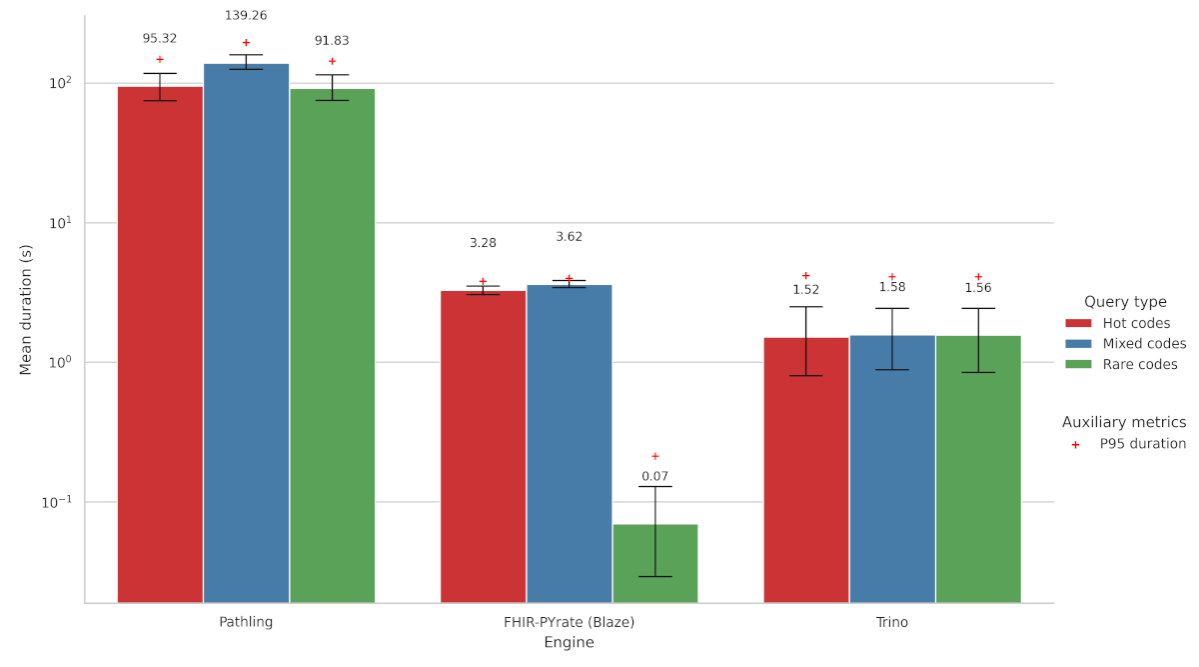
Log-scaled mean execution time (lower is better) for counting “Observation” resources with common, rare, and either codes. Error bars indicate the 95% CIs of the mean.

Figure 12 shows execution times for count queries over hot, rare, and mixed “Observation” codes. The selected rare codes accounted for 0.0006% (394/60,168,356) of the total observations, and the hot codes accounted for 15.3% (9,188,358/60,168,356). Pathling showed little sensitivity to code frequency. Hot- and rare-code workloads exhibited comparable runtimes, indicating similar end-to-end execution costs across skew levels, with mixed-code queries taking the longest. Blaze showed strong sensitivity to skew. Rare-code queries were completed substantially faster than hot- and mixed-code workloads. Mixed-code performance was close to hot-code performance. Trino exhibited no sensitivity to skew, with all workloads performing nearly identically.

## Discussion

There are at least 5 programming libraries that aim to implement analytical features using the FHIR Search API to retrieve data [8-11]. This is indicative not only of the increased use of FHIR as a format for conducting clinical research but also of an attempt at leveraging well-standardized interfaces: an FHIR server’s RESTful interface. However, the results of this benchmark show that frameworks purpose-built for analytics can execute common queries significantly faster. A key factor in this performance gap lies in the design differences between online transaction processing and online analytical processing systems. FHIR servers, typically designed for transactional workloads or accessing individual patient records, are not optimized for computationally intensive operations such as aggregations or large-scale data filtering and extraction. In contrast, Trino and Pathling leverage modern distributed data processing paradigms. These systems enable efficient execution plans by integrating data retrieval, computation, and caching mechanisms directly within their frameworks. Still, for the count workload, we found Blaze to outperform the tested alternatives for simpler queries and lower record counts.

For the aggregation and extraction scenario, one performance-limiting factor was the need to fetch the results from the FHIR server sequentially using pagination, as the FHIR Search specification does not define native aggregation operations. The FHIR-PYrate implementation, therefore, was required to retrieve all matching “Observation” resources and perform grouping client side in pandas. In contrast, both Trino and Pathling executed the aggregation directly within the query engine using optimized query planning. The FHIR-PYrate library does allow for parallel fetching of search results, but this requires specifying an expected beginning and end date based on which to partition the search queries. For these scenarios, using the FHIR bulk export standard may be a higher-performing alternative [28].

We limited the selection of queries to only those that could be implemented as a single FHIR Search query. This avoids adding too much postprocessing logic to the total execution time. However, this also revealed challenges in the FHIR Search expressiveness and implementation in the server [5]. For example, the diabetes query in the count scenario was initially supposed to count the number of distinct patients satisfying the criteria. This would require a count on the “Patient” resource type filtered using reverse chaining (“_has”) to only include those patients who were referenced in a diabetes “Condition” *and* in an “Encounter” in the specific date range within which the diagnosis was recorded. However, multiple “_has” parameters are processed independently, which would cause “Patient” resources with diabetes “Conditions” recorded as part of any “Encounter” to be counted as well. Furthermore, FHIR Search requires explicitly defining search parameters for nonstandard resource elements (such as for searching within extensions). Depending on the implementation, this also requires reindexing all resources before searches can be executed using custom parameters. Even the availability of standard search parameters varies between servers. Compared to FHIR Search, Trino is not as limiting in its expressiveness, as it is an American National Standards Institute SQL-compliant query engine. Similarly, Pathling is based on Apache Spark, where both SQL queries and a DataFrame API can be used to query the data.

Both Pathling and Trino access the same data source—FHIR resources encoded as Delta Lake tables—but use different implementations to query them. This is an example of storage-compute decoupling, where storage and computation engines can be scaled and replaced independently. This also allows for querying the data using any other framework that supports reading Delta Lake tables and makes it easier for users to use tools they are already familiar with. Despite these advantages, the transition from FHIR-native data structures to tabular formats introduces challenges: encoding FHIR resources in Delta Lake tables requires additional infrastructure and processing steps. This process not only incurs extract, transform, and load overhead but also necessitates standardization efforts as the schema of these tables is not standardized and currently bound to the implementation of the Pathling encoders. Standardizing the encoding of FHIR resources as “Open” table formats might, therefore, be a useful future endeavor to ensure interoperability for analytics [29]. Systematic performance benchmarking of alternative encodings of FHIR resources would be valuable future work as schema design choices can affect query performance across analytics engines.

The resource use measured when loading the Synthea resources from the disk to the FHIR servers and Delta Lake tables differed across the tested system. Using Pathling, the FHIR JSON files were encoded faster and ended up using less disk space than both the HAPI and Blaze FHIR servers. However, Pathling also used more CPU and memory during loading. The 2 FHIR servers were loaded using 1 transaction bundle per Synthea patient record, and even though the blazectl client concurrency was set to 32, the servers did not fully saturate the available CPUs. This could be a result of the transaction processing implementation limiting the maximum concurrency. In comparison, the Pathling server used bulk import functionality, which was likely less affected by database locks and similar bottlenecks necessary to ensure atomic transaction processing. If it were implemented in all evaluated systems, consistently using bulk import would make the results more comparable.

The limited cache sensitivity observed for count workloads suggests that these queries were dominated by full-table scans rather than metadata or plan reuse. In contrast, extraction workloads benefited from cache reuse in Pathling and Trino, likely reflecting reuse of Parquet or Delta Lake metadata, execution plans, and file system caches in Spark and MinIO. While Trino caches Delta Lake metadata by default (for 30 minutes), we did not enable file system caching, which might have shown a more significant impact on cache-warmed performance.

The HAPI FHIR server was unable to complete the hemoglobin query beginning with the lowest record counts. When attempting to run the count variant of the query, we observed a single PostgreSQL CPU max out until eventually the query was aborted with an “out of disk space” error. We believe this may be caused by the way in which multiple OR-combined code-value-quantity searches are translated to SQL queries in the server.

The observed differences in the impact of data skew on the count performance highlight how engines respond differently to skew in predicate selectivity and result cardinality. Pathling’s and Trino’s insensitivity to code frequency suggests that performance is dominated by full-table scans, with limited benefit from selective predicates in the current execution model. In contrast, Blaze benefited substantially from highly selective predicates. Mixed-code queries remained dominated by high-frequency codes, explaining why their performance resembled hot-code workloads. These results demonstrate that realistic long-tail code distributions can affect engine performance and that benchmarks assuming uniform code distributions may overstate the performance of scan-dominated pipelines. The present experiment isolated skew in single-table predicate selectivity; join-key skew and shuffle skew in multi-table and multi-worker cohort queries remain important directions for future work.

All benchmarks were run on a single virtual machine, which means that both the benchmarked components and the code used to observe them were not isolated from each other. Additionally, all network communication occurred on the same local loop-back network interface, making the results less realistic as the impact of network latency was not measured. As modern data processing frameworks are capable of—and even optimized for—running analytical queries distributed across multiple compute nodes, how these findings generalize to such setups necessitates further exploration.

Future research should aim to establish a common set of benchmark scenarios tailored to health care analytics on FHIR. Drawing inspiration from benchmarks such as TPC-H [30], such a framework could provide a standardized methodology for evaluating diverse analytics engines and facilitate comparison across tools and architectures. This is particularly relevant with the new SQL-on-FHIR standard [7].

While our results show that FHIR Search performs poorly for analytical workloads dominated by large scans and aggregations, FHIR servers in general provide strengths that are critical in production environments: fine-grained access control; provenance and audit logging; transactional guarantees; and standardized functionality for searching, creating, updating, patching, and deletion. In many deployments, these features outweigh analytical throughput and justify the use of FHIR Search for patient-level queries and standardizable workflows [31]. Our findings, therefore, do not argue against FHIR servers as a primary clinical data interface but, rather, highlight their unsuitability as a sole back end for large-scale cohort analytics and population-level queries, where columnar analytics engines can provide substantial performance advantages.

The FHIR standard is primarily designed for interoperability and the exchange of health care data. In particular, while the REST-based FHIR Search API is useful for standard queries and retrieving specific patient records, it is generally not suitable for high-performance, complex analytics, making it necessary to move the data to a more robust analytical environment. Engines such as Trino and Pathling demonstrated substantial performance advantages for analytics scenarios.

## Abbreviations

CPU: central processing unit
FHIR: Fast Healthcare Interoperability Resources
REST: representational state transfer

## Data Availability

The datasets generated during this study are available in the Zenodo repository [32]. The analysis source code is available at GitHub [33].
